# Potential Role of Activating Transcription Factor 5 during Osteogenesis

**DOI:** 10.1155/2016/5282185

**Published:** 2015-12-06

**Authors:** Luisa Vicari, Giovanna Calabrese, Stefano Forte, Raffaella Giuffrida, Cristina Colarossi, Nunziatina Laura Parrinello, Lorenzo Memeo

**Affiliations:** ^1^IOM Ricerca Srl, Via Penninazzo 11, 95029 Viagrande, Italy; ^2^Department of Experimental Oncology, Mediterranean Institute of Oncology, Via Penninazzo 7, 95029 Viagrande, Italy

## Abstract

Human adipose-derived stem cells are an abundant population of stem cells readily isolated from human adipose tissue that can differentiate into connective tissue lineages including bone, cartilage, fat, and muscle. Activating transcription factor 5 is a transcription factor of the ATF/cAMP response element-binding protein (CREB) family. It is transcribed in two types of mRNAs (activating transcription factor 5 isoform 1 and activating transcription factor 5 isoform 2), encoding the same single 30-kDa protein. Although it is well demonstrated that it regulates the proliferation, differentiation, and apoptosis, little is known about its potential role in osteogenic differentiation. The aim of this study was to evaluate the expression levels of the two isoforms and protein during osteogenic differentiation of human adipose-derived stem cells. Our data indicate that activating transcription factor 5 is differentially expressed reaching a peak of expression at the stage of bone mineralization. These findings suggest that activating transcription factor 5 could play an interesting regulatory role during osteogenesis, which would provide a powerful tool to study bone physiology.

## 1. Introduction

Human adipose-derived stem cells (hADSCs) are an alternative, accessible, and abundant source of stem cells readily isolated from adipose tissue that can differentiate* in vitro* into multiple lineages, including adipocytes, chondrocytes, osteocytes, neural-like cells, endothelial cells, and cardiomyocytes under lineage-specific culture conditions [1–9]. This tissue provides a potential adult stem cell reservoir for each individual representing an interesting resource for regenerative medicine [10–15].

The ability to isolate and expand the culture and differentiate the hADSCs* in vitro* into particular lineages provides the opportunity to study events associated with differentiation.

Activating transcription factor 5 (ATF5) is a member of the ATF/cAMP response element-binding protein (CREB) family, which includes a large group of basic leucine zipper (bZIP) proteins with different transcriptional regulatory functions [16]. ATF5 plays a pivotal role in promoting cell survival, differentiation, proliferation, and apoptosis [17].

Consistent with this, accumulating data have proven that ATF5 downregulation allows for differentiation in mature oligodendrocytes, neurons, and astrocytes [18–21]. Moreover ATF5 promotes proliferation of cerebral cortical neuroprogenitor cells and is required for terminal differentiation and survival of olfactory sensory neurons [22]. In addition several studies have previously demonstrated that ATF5 is highly expressed in a variety of tumors [23, 24].

ATF5 gene generates two transcripts, ATF5 isoform 1 (activating transcription factor 5, transcript variant 1: NM_012068.5) and ATF5 isoform 2 (activating transcription factor 5, transcript variant 2: NM_001193646.1). They differ only in their 5′-untranslated regions (UTRs) designated ATF5-5′ UTR*α* and ATF5-5′ UTR*β*; their coding regions are identical and originate the same 30-kDa protein [25]. The significance of these two transcripts is currently not clarified.

Although ATF5 is previously demonstrated to play a role in osteogenic differentiation [26, 27], it has never been described in detail. Furthermore, these two isoforms have never been investigated in osteogenesis.

In the present study we analysed the two ATF5 mRNA isoforms and protein to evaluate the modulation of their expression during different stages of bone formation. To this end, mRNAs and proteins were collected during the whole period to perform qRT-PCR and immunocytochemical analysis.

Our findings suggest that ATF5 mRNAs and protein present a different expression profile and provide new insights about ATF5 role in osteogenesis.

## 2. Materials and Methods

### 2.1. Isolation,* Ex Vivo* Expansion, and Characterization of Human Adipose-Derived Stem Cells

hADSCs were obtained from adipose tissue biopsies of the Pathology Unit at the Mediterranean Institute of Oncology (Viagrande, Italy) after informed consent. The adipose tissue was mechanically dissociated into smaller pieces and digested at 37°C with a collagenase I solution (Gibco, Thermo Fisher Scientific, Waltham, MA, USA). After two hours, the fragments were centrifuged, the floating fat was removed, and the remaining supernatant was filtered and centrifuged. To select adherent cells, the final pellet was resuspended in growth medium (ADSC basal medium, Lonza Group Ltd., Basel, Switzerland) supplemented with foetal bovine serum (FBS; Invitrogen, Thermo Fisher Scientific, Waltham, MA, USA), L-glutamine and gentamicin-amphotericin B (GA-1000, Lonza) and incubated overnight in 75 cm^2^ flasks at 37°C in a humidified atmosphere containing 5% CO_2_. The following day, nonadherent cells were removed. Selected hADSCs were maintained in 75 cm^2^ flask, and the medium changed every 3-4 days and expanded until 80–90% confluence.

hADSCs were characterized by flow cytometry analysis. 1 × 10^4^ cells/tube were stained with the following antibodies: CD45 FITC (clone J.33), CD34 PE (clone 581), CD73 PE (clone 581), CD90 FITC (clone F15.42.1.5), CD105 PE (clone 1G2), GlyA PE (clone 11E4B7.6), CD31PE (clone 1F11), CD117 PE (clone 104D2D1), CD271 FITC (clone ME20.4-1.H4), and respective isotopic controls according to manufacturer indications. All antibodies were purchased from Beckman Coulter (Milano, Italy), except CD271 that is provided by Miltenyi Biotec (Bologna, Italy).

### 2.2. *In Vitro* Osteogenic Differentiation of Human Adipose-Derived Stem Cells

For osteogenic differentiation, hADSCs were seeded at a density of 3,1 × 10^4^ cells/cm^2^ on collagen I coated plate in growth medium. After 24 hours, growth medium was replaced with osteogenic induction medium (Lonza) containing osteogenic basal medium (Lonza) supplemented with growth factors, dexamethasone, ascorbate, L-glutamine, penicillin/streptomycin, and *β*-glycerophosphate (Lonza). The medium was changed every 3-4 days. The osteogenic differentiation was observed during the whole period by microscopy. The osteogenic phenotype was confirmed by immunocytochemical analysis with specific markers and Alizarin Red S staining.

For immunocytochemical analysis the cells were seeded in 8-well BD Falcon culture slides (Corning Inc., Corning, NY, USA) at a density of 5000 cells per cm^2^ in ADSC-growth medium. After 48 hours the medium was removed, and the adherent cells were washed and fixed with paraformaldehyde 4% (PFA, Sigma Aldrich, Saint Louis, MO, USA) for 15 minutes. Subsequently, the cells were permeabilized and blocked. The primary incubation was performed, overnight at 4°C, with the following antibodies: rabbit anti-human ATF5 (1 : 500, Novus Biologicals, Littleton, CO, USA), rabbit anti-human osteopontin (1 : 250, Novus Biologicals), and mouse anti-human osteocalcin (1 : 200, Santa Cruz Biotechnology Inc., Dallas, TX, USA). After washing, slides were incubated with the appropriate secondary AlexaFluor 568 antibodies (Life Technologies Italia, Monza, Italy) for 2 hours at room temperature and the nuclei counterstained with DAPI. Afterwards they were mounted with fluorescent mounting medium Permafluor (Thermo Scientific). Digital images were acquired using a Leica DMI4000B fluorescence microscope (Leica Microsystems Srl, Milan, Italy) and cells count was performed by using CellProfiler [28].

For Alizarin Red S staining, cells were fixed with PFA 4% for 15 minutes and incubated with 2% Alizarin Red S solution for 5 minutes. After incubation the staining solution was removed and the culture slides were washed to eliminate excessive colour.

### 2.3. Total RNA Extraction and Reverse Transcription

Total RNA was extracted from hADSCs during the whole period with the RNeasy Mini Isolation Kit (Qiagen, Valencia, CA, USA). RNA purity was calculated measuring the ratio of the absorbance at 260 and 280 nm and considering 1.8–2.0 as admissible range of ratios for pure RNAs. RNA quality was determined by Agilent 2100 Bioanalyzer RNA assays (Agilent technologies, Santa Clara, CA, USA) and by calculating the ratio of the 28S and 18S ribosomal RNA intensity peaks. Total RNA was stored at −80°C.

RNA samples (1,0 *μ*g) were reverse-transcribed by using the High-Capacity cDNA Reverse Transcription (Applied Biosystems, Thermo Fisher Scientific, Waltham, MA, USA) according to manufacturer's protocol. RT products were stored at −20°C.

### 2.4. Real-Time RT-PCR

Target mRNAs concentration was assessed by quantitative real-time PCR (qRT-PCR) on Applied Biosystem 7900HT fast real-time PCR system using comparative Ct method with fast SYBR Green chemistry (Applied Biosystem). PCR primers were designed using Primer BLAST [29] to specifically recognize selected transcripts by targeting exon-exon junctions and tested for off-targeting using human RefSeq database [30]. Both human glyceraldehyde-3-phosphate dehydrogenase and tubulin beta were used as endogenous controls. Isoforms specific ATF5 primer pairs were designed to recognize unique regions in 5′ UTRs. Run-related transcription factor 2 (RUNX2), OSTERIX, and alkaline phosphatase (ALPL) primers were used to evaluate osteogenic commitment. Each molecular endpoint was tested in triplicate. Relative quantitation of mRNA expression has been evaluated using the ΔΔct method with the proliferation mRNA level used as reference. Statistical analysis of ATF5 expression levels in temporal groups has been performed using the analysis of variance.

The following primers were used to perform qRT-PCR: ATF5_1 fw: CAGGAAATTCTGCAAGCAAGGAA; ATF5_1 rev: CGGCGACACTCTTCCCTCTG; ATF5_2 fw: TGTCCTCGGATCACAGTCTCT; ATF5_2 rev: AAGTGGAAGACTCCATGGCTG; OSTERIX fw: TGCTTGAGGAGGAAGTTCACTATG; OSTERIX rev: TGCCCAGAGTTGTTGAGTCC; ALPL fw: GACCCTTGACCCCCACAAT; ALPL rev: CGCCTCGTACTGCATGTCCCCT; RUNX2 fw: GGAGTGGACGAGGCAAGAGTTT; RUNX2 rev: AGCTTCTGTCTGTGCCTTCTGG; GAPDH fw: GCTCTCCAGAACATCATCCCTGCC; GAPDH rev: GCGTTGTCATACCAGGAAATGAGCTT; *β*-TUB fw: GCGCATTCCAACCTTCCAG; *β*-TUB rev: CCCAGAACTTGGCACCGAT.

### 2.5. Western Blot

Cell pellets were homogenized with RIPA lysis buffer (1 : 5 w/v). For western blot quantification, 40 *μ*g of protein was separated on a precast 4–20% trisglycine gel (Thermo Scientific, Rockford, IL, USA) and transferred to a nitrocellulose membrane. After overnight blocking at 4°C with 5% nonfat dry milk, membranes were incubated for 4 h with rabbit anti-human ATF5 primary antibody (Novus Biologicals, Catalog number NBP2-15500; dilution 1 : 1000). Then, membranes were washed and incubated for 1 h with peroxidase-conjugate goat anti-rabbit secondary antibody (Thermo Scientific group; Catalog number 1858415; dilution 1 : 6000). Peroxidase activity was developed by enhanced chemiluminescent substrate (Pierce Biotechnology Inc., Thermo Scientific group; Catalog number 34075) and visualized by autoradiography. Then, the protocol was repeated for quantification of actin, using a rabbit anti-actin primary antibody (Santa Cruz, Catalog number SC130657; dilution 1 : 500) followed by a goat anti-rabbit secondary antibody (Pierce Biotechnology Inc., Thermo Fisher Scientific, Waltham, MA, USA; Catalog number 1858413; dilution 1 : 5000). Density values relative to all proteins were normalized to actin levels measured in the same membrane.

## 3. Results

### 3.1. hADSCs Differentiate in Osteocytes in Presence of Osteogenic Inductive Factors

**Figure 1:**
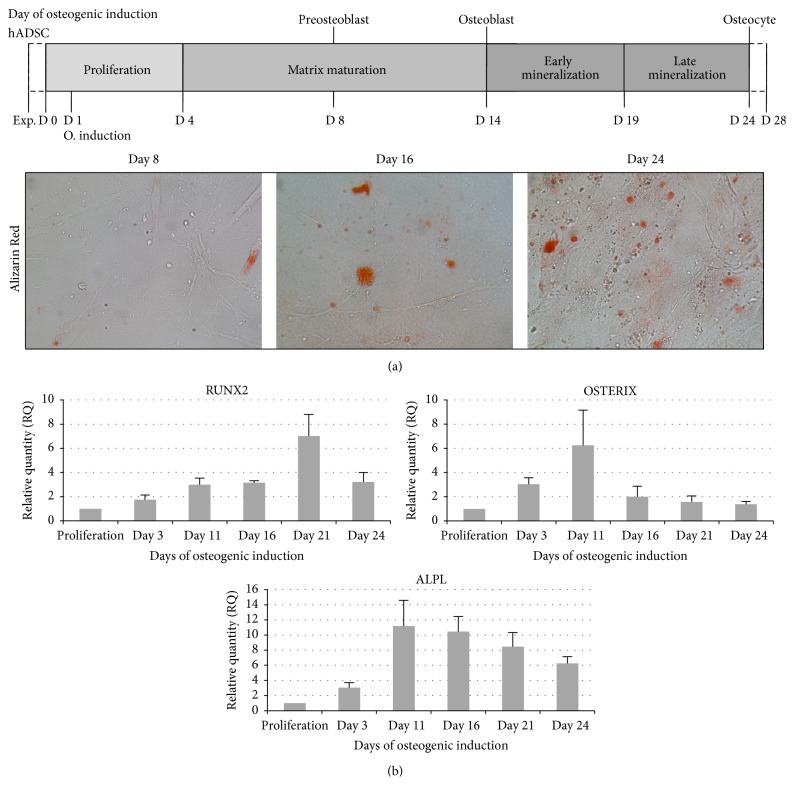
(a) Alizarin Red S staining of hADSCs during osteogenic differentiation. Mineralization of the extracellular matrix with the presence of calcium precipitates was visualized by staining with Alizarin Red S at days 8, 16, and 24. (b) Gene expression levels during osteogenic differentiation of hADSCs. Relative quantification of mRNA level assessed by qRT-PCR for RUNX2, OSTERIX, and ALPL after 3, 11, 16, 21, and 24 days of osteogenic differentiation. Proliferation group is used as reference for quantitation.

hADSCs derived from three different cell lines at passage 3 were cultured for 24 days in the presence of osteogenic medium. Alizarin Red S staining was used to examine the differentiation of hADSCs into osteocytes. After 8, 16, and 24 days of culture, hADSCs produced a densely mineralized extracellular matrix, followed by calcium precipitates within the cytoplasm. In particular Alizarin Red staining showed that cell calcium content increased over time; in fact, greater amount and size of calcium stores within the cytoplasm were observed (Figure 1(a)).

To confirm the osteogenic commitment of hADSCs we performed qRT-PCR analysis after 3, 11, 16, 21, and 24 days of osteogenic induction with the transcription factors RUNX2 and OSTERIX and the bone related gene ALPL (Figure 1(b)). The gene expression was analyzed in at least three different adipose-derived cell lines to compensate for the biological variance. qRT-PCR analysis of RUNX2, the central control gene within the osteoblast phenotype, showed that its expression level increased during osteogenic differentiation reaching a peak at day 21 according to what was previously reported [31]. The ALPL enzyme, an important component of osteogenesis, displayed a typical peak prior to mineralization (day 11), as shown in Figure 1(b).

The expression pattern of the OSTERIX gene was very similar to that of ALPL. OSTERIX, a marker of committed osteoprogenitors, was significantly enhanced after day 3 of osteogenic induction reaching a peak at day 11.

### 3.2. ATF5_1 and ATF5_2 Expression Levels Show a Peak at the Bone Mineralization Stage

To evaluate the expression levels of the two ATF5 isoforms (ATF5_1 and ATF5_2) during osteogenic differentiation we performed qRT-PCR analysis.

**Figure 2:**
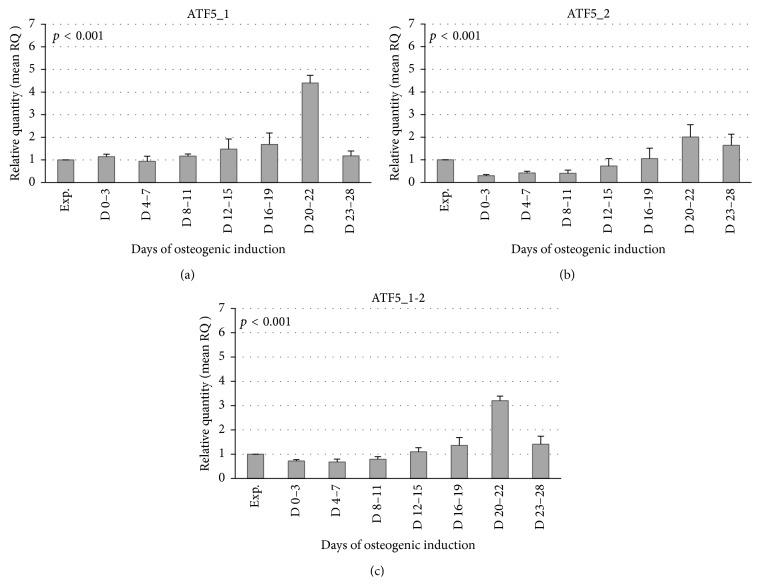
ATF5 expression levels during osteogenic differentiation of hADSCs. (a, b, c) Relative quantification of mRNA levels assessed by real-time PCR for ATF5 isoforms, conducted on RNA from hADSCs, either undifferentiated (proliferation) or differentiated (D 0–D 28). Proliferation group is used as reference for quantitation. The analysis of variance of both ATF5 isoforms relative expression levels indicates a statistically significant modulation during osteogenic differentiation (*p* < 0.001).

For qRT-PCR analysis we divided the days of osteogenic differentiation into 8 groups (proliferation, D 0–3, D 4–7, D 8–11, D 12–15, D 16–19, D 20–22, and D 23–28). The mean relative expression of ATF5 isoforms in each interval is reported in Figure 2. Proliferation group is used as reference for quantitation.

The results of qRT-PCR showed that the expression of ATF5_1 was unaltered between day 0 and day 7 and gradually increased until day 22 (approximately 3, 5 times), successively showing a threefold decrease at day 28 (Figure 2(a)).

The expression of ATF5_2 decreased during the first 15 days of osteogenic differentiation and reached a peak at days 20–22, decreasing again successively (Figure 2(b)). When both the isoforms were considered together (ATF5_1-2, average of the two ATF5 isoforms mRNAs), our data showed that after an initial decrease from day 0 to day 11 ATF5_1-2 levels increased with a peak at days 20–22 and then decreased approximately about 2 times (Figure 2(c)). Statistical analysis of ATF5 expression levels in temporal groups indicates a significant modulation of both isoforms during differentiation (*p* < 0.001 for both isoforms and for their averaged relative quantitation).

In summary our results showed that while ATF5_1 and ATF5_2 mRNAs expression profile seems to differ in the early stages of osteogenic induction (proliferation, matrix maturation, and early-mineralization stages), they exhibit the same expression peak in the stage of late bone mineralization (D 20–22), as also confirmed by the ATF5 protein expression. After D 23–28 days, both ATF5 mRNA isoforms were downregulated.

The present study provides the first description of the expression levels of the two ATF5 isoforms during osteogenic differentiation.

### 3.3. ATF5 Protein Is Expressed in the Bone Mineralization Stage during Osteogenic Differentiation

To evaluate ATF5 protein expression, cells growth in osteogenic medium after 3, 11, 16, 21, and 24 days was assessed by immunocytochemical analysis.

**Figure 3:**
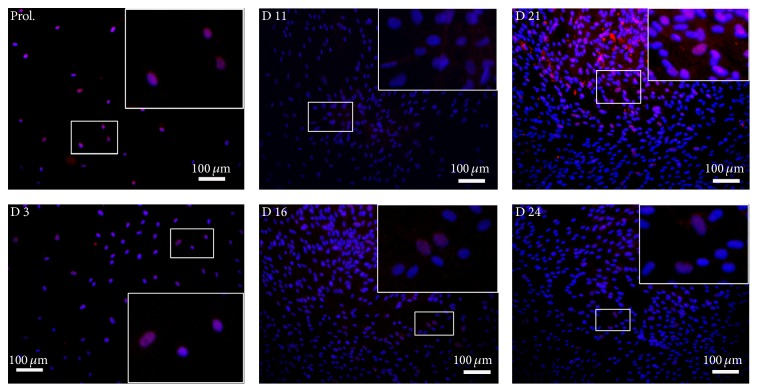
ATF5 during osteogenic differentiation. Immunocytochemical analysis of ATF5 at specific time points (proliferation, days 3, 11, 16, 21, and 24).

In accordance with the data of qRT-PCR, we found that ATF5 protein (Figure 3) had an expression peak (71,9%) during the stage of bone mineralization, day 21, decreasing successively after day 24 (5,9%) as shown in Table 1.

To evaluate a potential relationship between ATF5 and typical early/late bone mineralization markers, we performed an immunocytochemical analysis with osteopontin (OP, early osteogenic marker) and osteocalcin (OC, late osteogenic marker).

**Figure 4:**
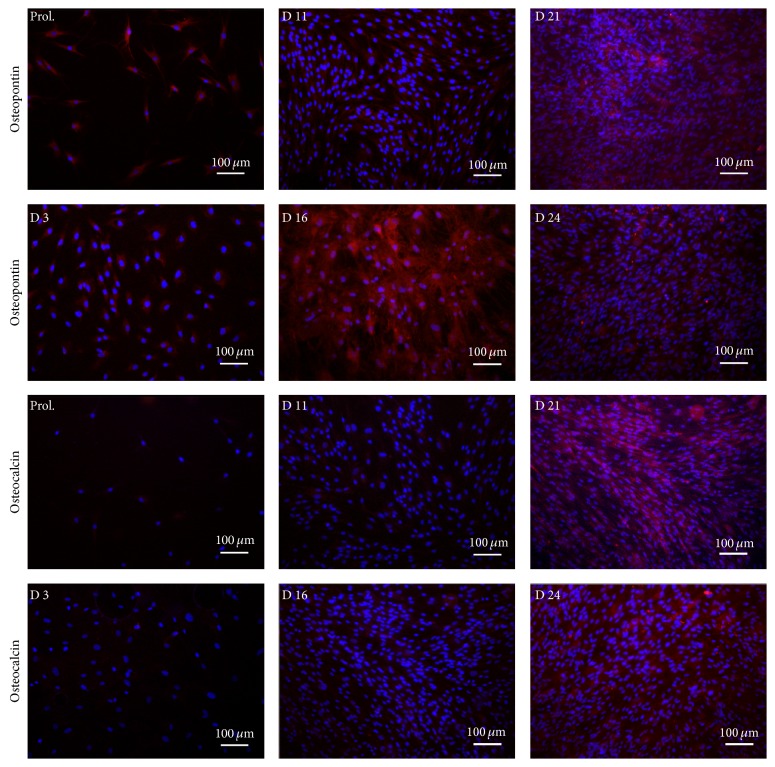
Early and late markers during osteogenic differentiation. Immunocytochemical analysis of OP and OC at specific time points (proliferation, days 3, 11, 16, 21, and 24).

Consistent with previous studies we observed that hADSCs had a basal OP expression during proliferation and the intensity increased remarkably at day 16 during bone mineralization; at day 21 a significative number of OP and OC positive cells were present (Figure 4).

### 3.4. Western Blot

**Figure 5:**
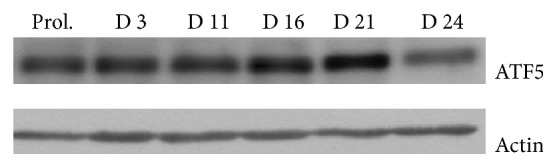
Western blot analysis, using human anti-ATF5 antibody, in hADSCs at specific time points (proliferation, days 3, 11, 16, 21, and 24).

In order to evaluate ATF5 levels during the different stages of osteogenic differentiation, we performed a western blot analysis at different time points: proliferation, days 3, 11, 16, 21, and 24. Our results show a low expression of ATF5 at early stages (proliferation, day 3 and day 11) with protein level increase from day 16 to day 21 and markedly reduced quantity during the later stage of osteogenic differentiation (day 24) (Figure 5). These data are in line with those obtained by cytochemical analysis.

## 4. Discussion

Osteogenesis is a complex process comprising various stages, including proliferation, condensation, differentiation, and activation of bone cells, which lead to the matrix maturation and successive mineralization. A great variety of molecules and pathways are required to induce the osteogenic process, including molecules belonging to the wingless-int (WNT), the bone morphogenetic protein (BMP), the hedgehog (HH), and the fibroblast growth factor (FGF) families [31–33].

During the last decades, the advancements in the use of MSCs in regenerative therapy have driven increasing attention on the molecular networks involved in bone formation process. Among these, Leong et al. [26, 27] reported that ATF5 expression decreased at day 28 of hADSCs osteogenic differentiation. In addition, they showed that knockdown of ATF5 with siRNA presented an increased sensitivity to osteogenic induction.

Our study provides the first description of temporal change of ATF5 isoforms expression during osteogenic differentiation. In particular, we observed that ATF5_1 and ATF5_2 reached a peak of expression at the stage of bone mineralization and subsequently decreased in the final stage (day 24), reaching almost the proliferation levels.

Although we do not have a mechanistic explanation for these findings, the present study could represent a first step elucidating the relationship between ATF5 and typical osteogenic related genes.

These results indicate that ATF5 could play a potential role in bone mineralization. Therefore, it would be reasonable to believe that it could operate together with the osteogenic differentiation genes previously described in our paper as OC and OP, or RUNX2, distal-less homeobox 5 (Dlx5), and bone sialoprotein (BSP) recently reported by Hagh et al. [34] that showed the same ATF5 trend.

Finally, functional studies are necessary to identify the transcriptional targets of this factor and the mechanisms by which its expression is regulated.

## 5. Conclusions

In conclusion, we have provided evidence that both ATF5_1 and ATF5_2 mRNA isoforms are potentially involved in the osteogenic induction of hADSCs* in vitro*, as demonstrated by the peaks of expression at the stage of bone mineralization. Furthermore, these preliminary data could suggest that ATF5 works together with specific osteogenic genes for the regulation of bone mineralization, providing new information about the two isoforms' involvement in osteogenic development.

## Figures and Tables

**Table 1:** Number and percentage of positive cells for ATF5, OP, and OC. Percentages of positive cells were calculated on the total amount of DAPI for each image (Figure 3).

	Prolif.			D 3			D 11			D 16			D 21			D 24		
	D	R	%	D	R	%	D	R	%	D	R	%	D	R	%	D	R	%
ATF5	31	29	93,5	76	53	69,7	309	1	0,3	511	100	19,6	814	585	71,9	589	35	5,9

OP	29	29	100	105	86	81,9	466	234	50,2	128	128	100	834	329	39,4	783	321	41

OC	23	5	21,7	136	2	1,5	356	140	39,3	811	265	32,7	750	248	33	805	331	41,1

## References

[1] Mailey B., Hosseini A., Baker J. (2014). Adipose-derived stem cells: methods for isolation and applications for clinical use. *Methods in Molecular Biology*.

[2] Baer P. C., Geiger H. (2012). Adipose-derived mesenchymal stromal/stem cells: tissue localization, characterization, and heterogeneity. *Stem Cells International*.

[3] Bassi G., Pacelli L., Carusone R., Zanoncello J., Krampera M. (2012). Adipose-derived stromal cells (ASCs). *Transfusion and Apheresis Science*.

[4] Bunnell B. A., Flaat M., Gagliardi C., Patel B., Ripoll C. (2008). Adipose-derived stem cells: isolation, expansion and differentiation. *Methods*.

[5] Locke M., Windsor J., Dunbar P. R. (2009). Human adipose-derived stem cells: isolation, characterization and applications in surgery. *ANZ Journal of Surgery*.

[6] Kern S., Eichler H., Stoeve J., Klüter H., Bieback K. (2006). Comparative analysis of mesenchymal stem cells from bone marrow, umbilical cord blood, or adipose tissue. *Stem Cells*.

[7] Puissant B., Barreau C., Bourin P. (2005). Immunomodulatory effect of human adipose tissue-derived adult stem cells: comparison with bone marrow mesenchymal stem cells. *British Journal of Haematology*.

[8] Gimble J. M., Guilak F. (2003). Differentiation potential of adipose derived adult stem (ADAS) cells. *Current Topics in Developmental Biology*.

[9] Zuk P. A., Zhu M., Ashjian P. (2002). Human adipose tissue is a source of multipotent stem cells. *Molecular Biology of the Cell*.

[10] Zuk P. A., Zhu M., Mizuno H. (2001). Multilineage cells from human adipose tissue: implications for cell-based therapies. *Tissue Engineering*.

[11] Tsuji W., Rubin J. P., Marra K. G. (2014). Adipose-derived stem cells: implications in tissue regeneration. *World Journal of Stem Cells*.

[12] Romagnoli C., Brandi M. L. (2014). Adipose mesenchymal stem cells in the field of bone tissue engineering. *World Journal of Stem Cells*.

[13] Kim E. H., Heo C. Y. (2014). Current applications of adipose-derived stem cells and their future perspectives. *World Journal of Stem Cells*.

[14] Konno M., Hamabe A., Hasegawa S. (2013). Adipose-derived mesenchymal stem cells and regenerative medicine. *Development Growth and Differentiation*.

[15] Gimble J. M., Katz A. J., Bunnell B. A. (2007). Adipose-derived stem cells for regenerative medicine. *Circulation Research*.

[16] Persengiev S. P., Green M. R. (2003). The role of ATF/CREB family members in cell growth, survival and apoptosis. *Apoptosis*.

[17] Wei Y., Ge Y., Zhou F. (2010). Identification and characterization of the promoter of human ATF5 gene. *Journal of Biochemistry*.

[18] Greene L. A., Lee H. Y., Angelastro J. M. (2009). The transcription factor ATF5: role in neurodevelopment and neural tumors. *Journal of Neurochemistry*.

[19] Angelastro J. M., Ignatova T. N., Kukekov V. G. (2003). Regulated expression of ATF5 is required for the progression of neural progenitor cells to neurons. *The Journal of Neuroscience*.

[20] Angelastro J. M., Mason J. L., Ignatova T. N. (2005). Downregulation of activating transcription factor 5 is required for differentiation of neural progenitor cells into astrocytes. *The Journal of Neuroscience*.

[21] Mason J. L., Angelastro J. M., Ignatova T. N. (2005). ATF5 regulates the proliferation and differentiation of oligodendrocytes. *Molecular and Cellular Neuroscience*.

[22] Wang S.-Z., Ou J., Zhu L. J., Green M. R. (2012). Transcription factor ATF5 is required for terminal differentiation and survival of olfactory sensory neurons. *Proceedings of the National Academy of Sciences of the United States of America*.

[23] Monaco S. E., Angelastro J. M., Szabolcs M., Greene L. A. (2007). The transcription factor ATF5 is widely expressed in carcinomas, and interference with its function selectively kills neoplastic, but not nontransformed, breast cell lines. *International Journal of Cancer*.

[24] Sheng Z., Ma L., Sun J. E., Zhu L. J., Green M. R. (2010). An activating transcription factor 5-mediated survival pathway as a target for cancer therapy. *Oncotarget*.

[25] Hatano M., Umemura M., Kimura N. (2013). The 5′-untranslated region regulates ATF5 mRNA stability via nonsense-mediated mRNA decay in response to environmental stress. *The FEBS Journal*.

[26] Leong D. T., Abraham M. C., Gupta A., Lim T.-C., Chew F. T., Hutmacher D. W. (2012). ATF5, a possible regulator of osteogenic differentiation in human adipose-derived stem cells. *Journal of Cellular Biochemistry*.

[27] Leong D. T., Abraham M. C., Chew F. T., Lim T. C., Hutmacher D. W. (2007). ATF5, a possible regulator of osteogenic differentiation in adult mesenchymal stem cells. *Journal of Stem Cells Regenerative Medicine*.

[28] Carpenter A. E., Jones T. R., Lamprecht M. R. (2006). CellProfiler: image analysis software for identifying and quantifying cell phenotypes. *Genome Biology*.

[29] Ye J., Coulouris G., Zaretskaya I., Cutcutache I., Rozen S., Madden T. (2012). Primer-BLAST: a tool to design target-specific primers for polymerase chain reaction. *BMC Bioinformatics*.

[30] Pruitt K. D., Brown G. R., Hiatt S. M. (2014). RefSeq: an update on mammalian reference sequences. *Nucleic Acids Research*.

[31] Kirkhamand G. R., Cartmell S. H. (2007). Genes and proteins involved in the regulation of osteogenesis. *Topics in Tissue Engineering*.

[32] Milat F., Ng K. W. (2009). Is Wnt signalling the final common pathway leading to bone formation?. *Molecular and Cellular Endocrinology*.

[33] Zaidi M. (2007). Skeletal remodeling in health and disease. *Nature Medicine*.

[34] Hagh M. F., Noruzinia M., Mortazavi Y. (2015). Different methylation patterns of RUNX2, OSX, DLX5 and BSP in osteoblastic differentiation of mesenchymal stem cells. *Cell Journal*.

